# Effect of Bonding Strength on Electromigration Failure in Cu–Cu Bumps

**DOI:** 10.3390/ma14216394

**Published:** 2021-10-25

**Authors:** Kai-Cheng Shie, Po-Ning Hsu, Yu-Jin Li, K. N. Tu, Chih Chen

**Affiliations:** 1 Department of Materials Science and Engineering, National Yang Ming Chiao Tung University, Hsinchu 30010, Taiwan; alex911666.mse06g@g2.nctu.edu.tw (K.-C.S.); seraph8938.mse03g@g2.nctu.edu.tw (P.-N.H.); a0481518.mse04g@g2.nctu.edu.tw (Y.-J.L.); kntu@ucla.edu (K.N.T.); 2 Department of Materials Science and Engineering, National Chiao Tung University, Hsinchu 30010, Taiwan; 3 International College of Semiconductor Technology, National Yang Ming Chiao Tung University, Hsinchu 30010, Taiwan; 4 International College of Semiconductor Technology, National Chiao Tung University, Hsinchu 30010, Taiwan

**Keywords:** electromigration, Cu–Cu direct bonding, three-dimensional integrated circuits (3D ICs)

## Abstract

In microelectronic packaging technology for three-dimensional integrated circuits (3D ICs), Cu-to-Cu direct bonding appears to be the solution to solve the problems of Joule heating and electromigration (EM) in solder microbumps under 10 μm in diameter. However, EM will occur in Cu–Cu bumps when the current density is over $10^{6}\;A/{\mathrm{cm}}^{2}$. The surface, grain boundary, and the interface between the Cu and TiW adhesion layer are the three major diffusion paths in EM tests, and which one may lead to early failure is of interest. This study showed that bonding strength affects the outcome. First, if the bonding strength is not strong enough to sustain the thermal mismatch of materials during EM tests, the bonding interface will fracture and lead to an open circuit of early failure. Second, if the bonding strength can sustain the bonding structure, voids will form at the passivation contact area between the Cu–Cu bump and redistribution layer (RDL) due to current crowding. When the void grows along the passivation interface and separates the Cu–Cu bump and RDL, an open circuit can occur, especially when the current density and temperature are severe. Third, under excellent bonding, when the voids at the contact area between the Cu–Cu bump and RDL do not merge together, the EM lifetime can be more than 5000 h.

## 1. Introduction

Due to the ending of Moore’s law on the miniaturization of two-dimensional integrated circuits (2D IC), 3D ICs through chip stacking technology is the most promising way to continuously improve the chip performance [1,2,3]. In 3D ICs, because there are many reliability issues in shrinking solder joints [4,5,6,7,8,9,10], Cu-to-Cu direct bonding technology has been introduced to scale down the diameter of joints [11,12,13,14]. Below, we shall use Cu–Cu to represent Cu-to-Cu.

On Cu–Cu direct bonding, a low thermal budget in lowering the bonding temperature to 250 °C and bonding time to 1 min are the key requirements to be adopted in advanced electronic packaging for high-density interconnects (<10 μm bump diameter) [15,16,17,18,19,20]. However, with the high current stressing in Cu–Cu interconnects, EM takes place again in interconnects, as well as in lines of the redistribution layer (RDL) [21,22,23,24].

The EM damages of Cu–Cu interconnects include void formation at Cu/SiO_2_ and Cu/adhesion layer interfaces and Cu–Cu bonding interfaces [22,25]. The reason of void formation at interfaces can be referred from the EM of Cu damascene structures [26,27,28,29,30], but voids at the bonding interface have not been well discussed. Although there have been previous studies of EM in Cu–Cu interconnects [22], the effect of bonding strength has not been mentioned.

In this study, Cu–Cu direct bonding microbumps were fabricated [18] to perform EM tests. The bonding strength was measured by the die pull test, as well as by varying the pressure and time in thermal compression bonding. Void formation was studied by both destructive and nondestructive observations. Finite element analysis (FEA) was adopted to realize the current density and stress distribution. According to these analysis results, EM failure mechanisms of Cu–Cu bumps can be systemically studied.

## 2. Materials and Methods

### 2.1. Sample Fabrication

**Figure 1:**
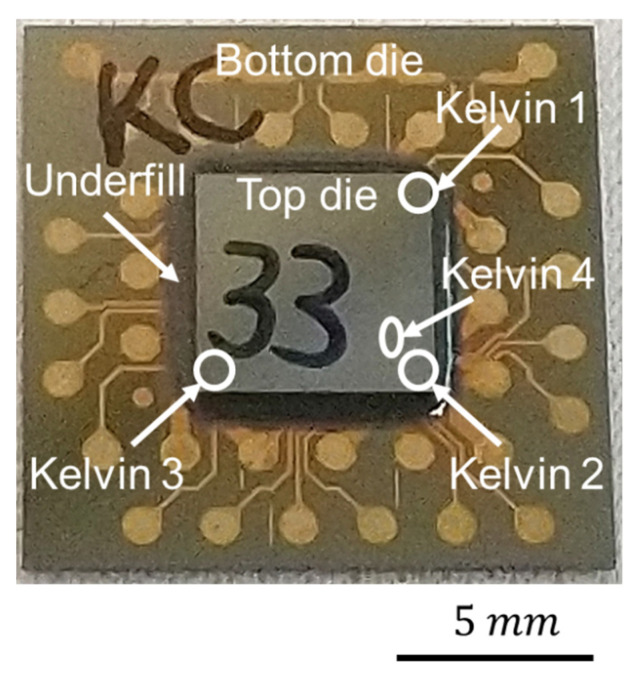
The test vehicle of Cu–Cu direct bonding and EM test. After the bonding process, underfill was dispensed into the chip. The sites of electrical test structures are marked on the chip.

The sample for the EM test is shown in Figure 1. A top die was bonded to a bottom die through instant Cu–Cu direct bonding [18,31]. The method of electroplating nanotwinned Cu (nt-Cu) [32,33] was adopted to fabricate nt-Cu microbumps because nt-Cu columnar grains have a (111) surface for low-thermal-budget bonding [15] and good mechanical properties [34,35,36,37]. However, the structure of passivation (PSV) opening led to some randomness of nt-Cu columnar grains. More sample fabrication details are in Reference [18]. In this study, the bonding temperature was 300 °C. The two conditions of bonding pressure and time were 90 MPa/30 s and 31 MPa/10 s.

**Figure 2:**
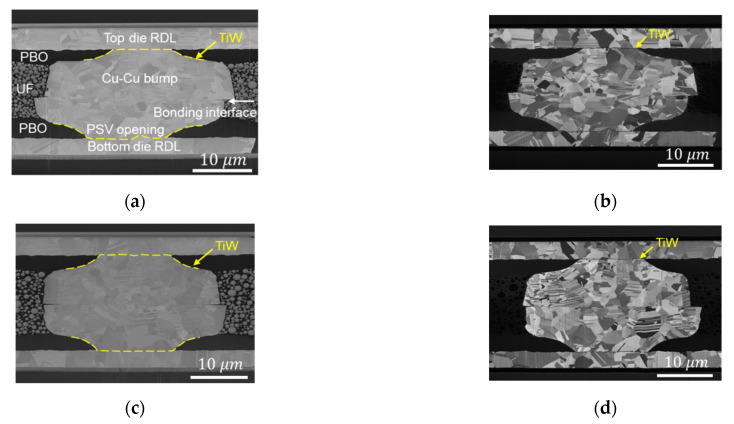
The cross-sectional images of as-fabricated Cu–Cu bumps. (**a**) Electron image and (**b**) ion image of 300 °C/90 MPa/30 s. (**c**) Electron image and (**d**) ion image of 300 °C/31 MPa/10 s.

The cross-sectional images of two types of Cu–Cu bumps are shown in Figure 2. The top and the bottom die RDLs connect with a Cu–Cu bump, and the adhesion layer of TiW between them is pointed out by the yellow dashed lines in Figure 2a,c. The photosensitive polybenzoxazole (PBO) cover on the RDLs serves as a passivation layer. A PSV opening was performed through lithography to define the pattern of Cu–Cu bumps. In order to protect the Cu–Cu bumps from oxidation, underfill (UF) was dispensed in the chip, which can be seen around the top die in Figure 1, and was filled well around Cu–Cu bumps in Figure 2a,c.

### 2.2. Test Vehicle of Resistance Measurement

**Figure 3:**
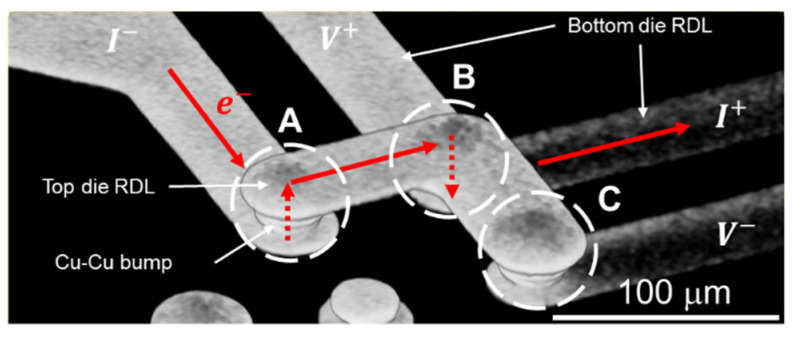
Nondestructive observation of Kelvin bumps. The resistance of the corner bump, named Kelvin bump B, can be measured. Kelvin bump A is under the same current stress during the EM test. Kelvin bump C connects with the $V^{-}$ probe pad.

Kelvin structures were designed in chips to perform EM tests in Figure 1. Kelvin structures 1~3 were located at the corners of the top die, and a 3D image was observed by the 3D X-ray machine (Carl Zeiss Co. Ltd., Oberkochen, Germany), as shown in Figure 3. The Kelvin structure was composed of three Cu–Cu bumps, named A, B, and C, and RDLs. Current was applied by a power supply and the electron flow is marked by red arrows, so Kelvin bumps A and B were under high current stressing during the EM test. However, only the resistance of Kelvin bump B could be measured.

### 2.3. Electromigration Test

The diameter of the bonding interface was 30 μm, and that of the PSV opening was 14 μm. The pitch of Cu–Cu bumps was 80 μm. The height of Cu–Cu bumps was 14 μm. The width of the RDL was 45 μm, and the thickness was 3 μm. However, the actual dimension mentioned above might vary slightly because of the sample fabrication processes. The current of the EM test was 1.5 A, and the chip was put on a hotplate (Yotec Instruments CO., LTD, Hsinchu City, Taiwan) at 150 °C. The average current density at the bonding interface was $2.0\times 10^{5}\;A/{\mathrm{cm}}^{2}$, and at the PSV opening, it was $9.8\times 10^{5}\;A/{\mathrm{cm}}^{2}$. In the RDL, the average current density was $1.1\times 10^{6}\;A/{\mathrm{cm}}^{2}$. Thus, EM damages were expected to occur faster near the PSV opening and RDLs.

As Joule heating occurred significantly during EM tests [38], the actual temperature was measured through the method of the temperature coefficient of resistance (TCR). The measured temperature was different slightly from chip to chip, and the maximum value due to Joule heating was close to 50 °C. The resistance of Kelvin bump B was recorded every 30 s during the EM test, and the criteria of EM failure were defined as 20% resistance change.

The microstructure of EM damages in Kelvin bumps was observed by the nondestructive observation of 3D X-rays (Carl Zeiss Co. Ltd., Oberkochen, Germany), and the destructive observation of Scanning electron microscope (SEM) and dual-beam focused ion beam (DB-FIB) (Field Electron and Ion Company, Hillsboro, Oregon, USA). A model of a single microbump was made to carry out FEA. The current density distribution could determine the current crowding effect. The distribution of tensile stress along the z-axis could confirm the required bonding strength to avoid an open-circuit. Three chips of each bonding condition were fabricated without UF dispensing to perform the pull test, so the average bonding strength of each Cu–Cu bump could be measured. A table listing the pull test of bonding strength is shown later.

## 3. Results and Discussion

### 3.1. As-Prepared Sample

Cross-sectional images of the microstructure of bonding conditions at 300 °C/90 MPa/30 s and at 300 °C/31 MPa/10 s are shown in Figure 2a–d, respectively. The electron images of Figure 2a,c show the void-free bonding interface. The ion images of Figure 2b,d show the microstructure of Cu grains. Due to the high bonding temperature and pressure, even for such a short bonding time, recrystallization of nt-Cu columnar grains occurred during the bonding process. However, Cu grains did not have enough time for grain growth, and the bonding interface was straight and clear across the Cu–Cu bumps. More residual nt-Cu columnar grains are shown in Figure 2d because of the lower bonding pressure and time.

### 3.2. Electromigration Tests

**Figure 4:**
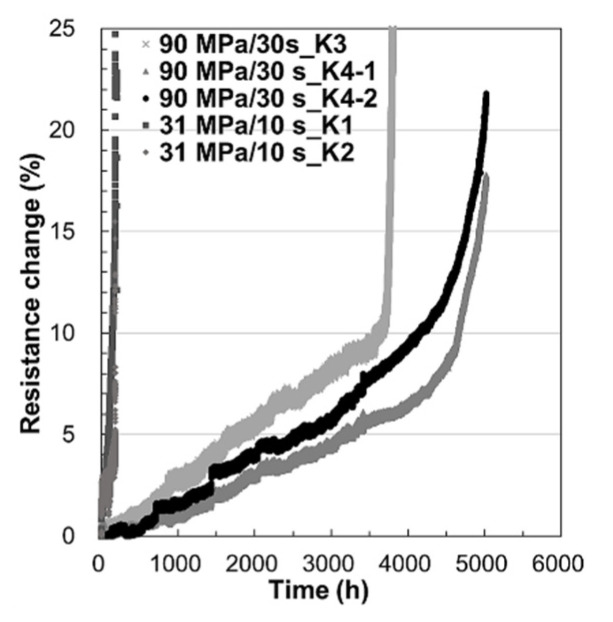
The lifetime of EM test. For the bonding condition 300 °C/90 MPa/30 s, the lifetime is longer than 3500 h. For the bonding condition 300 °C/31 MPa/10 s, the lifetime is longer than 170 h.

The resistance change during the EM test is shown in Figure 4. The EM lifetime of the bonding condition at 90 MPa/30 s was at least longer than 3500 h, while that of the bonding condition at 31 MPa/10 s was only 170 h. The profiles of resistance change of 90 MPa/30 s started from a steady increase, and then increased dramatically after it reached 10%. This phenomenon is similar to the EM of damascene Cu interconnects [28]. In the early part of the resistance steady increase, voids form at the bonding interface and continuously increase. Once the void reaches the critical dimension, resistance will increase abruptly [39]. For the EM of solder bumps, the same issue of the change in resistance profile has been discussed. It is because current crowding enhances the growth rate of pancake-type voids below the contact interface, but the resistance does not increase abruptly before the open circuit [40]. Below, the EM failure modes of Cu–Cu bumps are discussed.

### 3.3. EM Failure at the Bonding Interface

**Figure 5:**
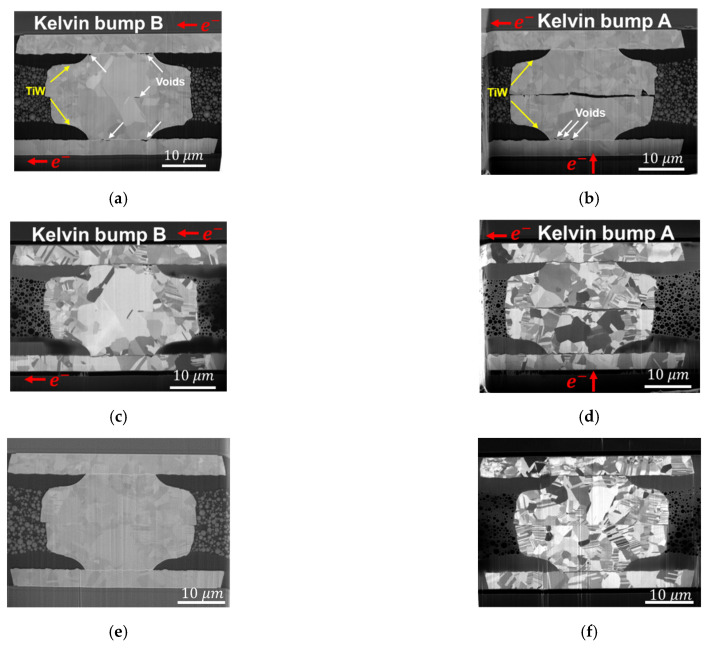
Destructive observation of the 31 MPa/10 s_K1 bumps. (**a**,**b**) Electron images of Kelvin bumps B and A, respectively. Voids are observed at the sites of current crowding. The voids form at the side of electron current passing through the TiW adhesion layer. In addition, the crack at the bonding interface of Kelvin bump A has caused an open circuit. (**c**,**d**) Ion images of Kelvin bumps B and A, respectively. In Kelvin bump B, grain growth across the bonding interface might be caused by the significant Joule heating before the open circuit of Kelvin bump A. (**e**,**f**) Electron image and ion image of a dummy bump, respectively.

The cross-sectional images of 31 MPa/10 s_K1 bumps are shown in Figure 5. Many tiny voids are found to be located at the PSV openings, which are indicated by the white arrows in the electron images of Figure 5a,b, in Kelvin bumps A and B, respectively. As vacancies cannot pass through the TiW layers [41], they accumulate to form voids on the TiW layers. According to a previous study of solder bumps [40], the sites of void formation are strongly related to current crowding, so the voids in Figure 5a,b are located at the corners between the PSV opening and RDL. 

In Figure 5a, a void in the middle of the bonding interface of Kelvin bump B may be caused by grain growth to eliminate the bonding interface. As the as-bonded bonding interface is similar to a grain boundary with a high vacancy concentration, the vacancies might congregate to form a void, and the void might undergo ripening [42,43]. The big grains are shown in Figure 5c. A large grain has grown across the center of the bonding interface, and some small grains are separated at the two sides of the Cu–Cu bump. 

In Figure 5b,d, a large crack is shown along the bonding interface, so the circuit of the Kelvin bump is open. Figure 5e,f show the electron image and ion image of the dummy bump, respectively. The dummy bump does not undergo high current stressing but is located in the same chip and on the hotplate for the same time. There is no void in Figure 5e. The bonding interface maintains a straight line in Figure 5f, and many nt-Cu columnar grains remain. Compared with the as-fabricated Cu–Cu bump in Figure 2d, small recrystallization grains grow during the EM test. However, the grain size of Figure 5f is smaller than that of Figure 5c,d because of the lack of Joule heating in the EM test.

### 3.4. EM Failure at the Passivation (PSV) Opening

**Figure 6:**
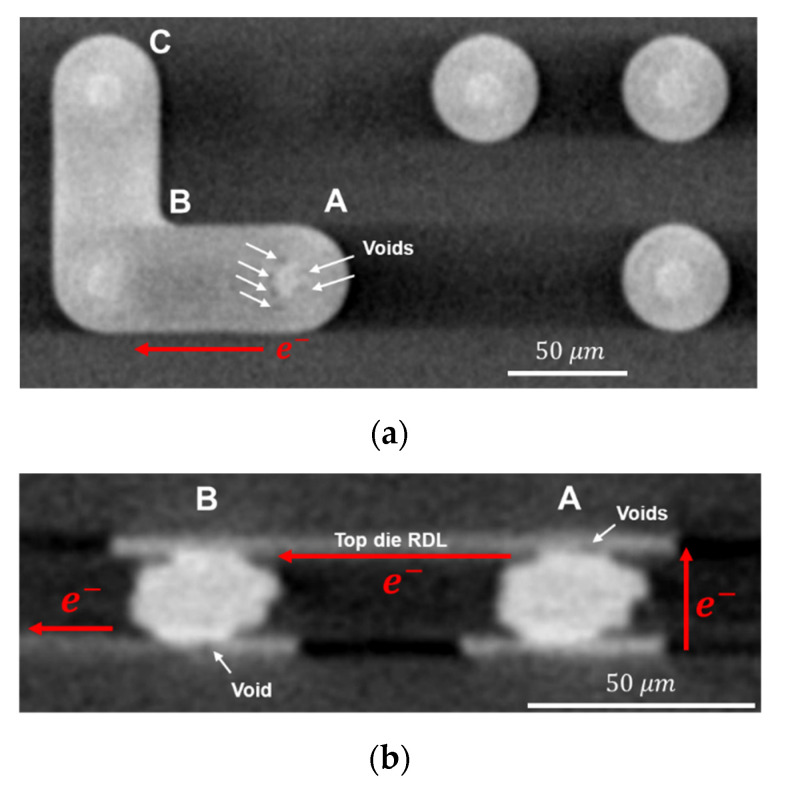
Nondestructive observation of 90 MPa/30 s_K3 bumps. (**a**) Computed tomography of the top die RDL. The voids form at the top die RDL of Kelvin bump A. (**b**) Computed tomography of the cross-section along the current direction in Kelvin bumps. The voids are observed at the top die RDL of Kelvin bump A and at the bottom die RDL of Kelvin bump B.

To realize voids distribution of the EM test, the Cu–Cu bump of 90 MPa/30 s_K3 was observed by 3D X-ray tomography, which is shown in Figure 6. The resolution of the 3D X-ray was 0.8 μm. The final resistance change of 90 MPa/30 s_K3 reached 115%, so the void size was large enough to be recognized. The computed tomography (CT) of the top die RDL is shown in Figure 6a. Due to the electron current direction and the TiW layer, voids can be recognized easily at the top die RDL of Kelvin bump A. Most of the voids distributed at the side close to Kelvin bump B of the PSV opening, and some voids were at the opposite PSV opening. There was no void at the top die RDL of Kelvin bump B. 

**Figure 7:**
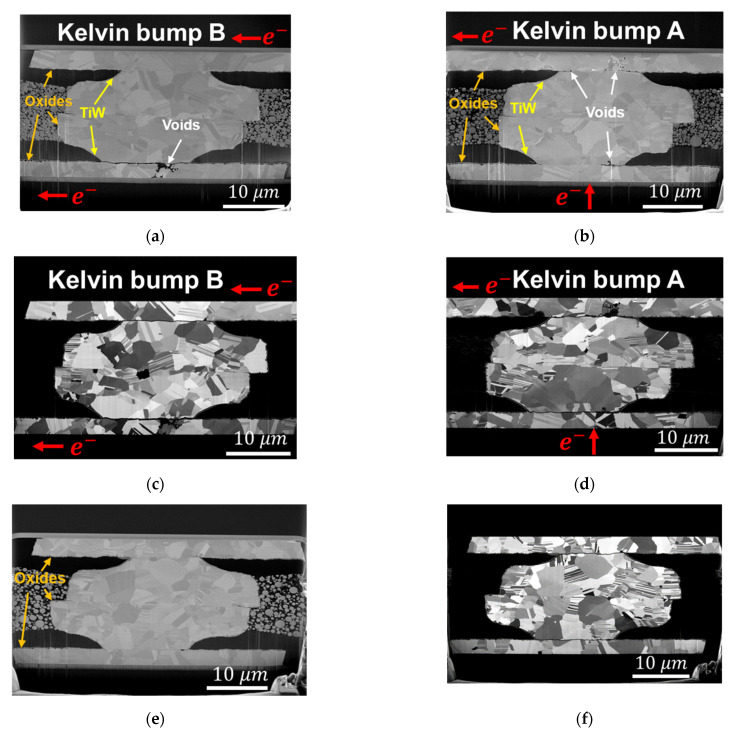
Destructive observation of 90 MPa/30 s_K3 bumps. (**a**,**b**) Electron images of Kelvin bumps B and A, respectively. Voids are located at the top die RDL of Kelvin bump A and the bottom die RDL of Kelvin bump B, which is the same as the nondestructive observation results. The voids at the lower part of Cu–Cu bump A can be seen in (**b**). The voids form at the side of electron current passing through the TiW layer. (**c**,**d**) Ion images of Kelvin bumps B and A, respectively. The voids caused by the EM are along the grain boundary and TiW layer at the PSV opening. (**e**,**f**) Electron image and ion image of dummy bumps, respectively. Copper oxide can be found at the surface of Cu–Cu bumps and RDLs.

The CT of the cross-section along the electron current flow direction in Kelvin bumps is shown in Figure 6b. The void of Kelvin bump A was located in the top die RDL, and the void of Kelvin bump B was located in the bottom die RDL. Due to the limitation of the 3D X-ray resolution, if the void size is too small in CT, it is difficult to recognize. The left part of the voids in the top die RDL of Kelvin bump A in Figure 6a is clear, but they are ambiguous in Figure 6b. Therefore, the void shape must be a plane-shape in the top die RDL and close to the PSV opening. On the contrary, the right-hand-side part of the voids in the top die RDL of Kelvin bump A tend to be a long-shaped void across the height of the RDL. It can also be proven in Figure 7 of destructive observation, as shown below.

Figure 7 shows the electron and ion cross-sectional images of the same Kelvin bump in Figure 6, but a more precise microstructure of the cross-sectional images can be observed in Figure 7. In Figure 7a, a narrow void is shown under the TiW layer at the bottom of Kelvin bump B, which may have caused the open circuit of the Kelvin structure, and the resistance change is 115% after 4035 h of EM stressing time. The Kelvin 3 structure is shown at the lower left corner of the chip, which is shown in Figure 1. Owing to such a long EM stressing time and also close to the edge of the top die, there is a layer of oxide covering the surface of the Kelvin bump and dummy bump, which is shown in Figure 7a,b,e. As Cu oxide has a poor adhesion to Cu, there are some voids between the oxide and Cu, as shown in Figure 7e of the dummy bump. More serious void formation in Figure 7a,b might be caused by EM in driving Cu surface diffusion, and Joule heating enhances the oxidation rate. However, the oxide thickness does not show a big difference between the Kelvin bump and dummy bump. Moreover, the voids of Kelvin bumps between the oxide and the bottom die RDLs are more serious than those of the top die RDL. It might be the short-length effect in EM [28]. 

During the EM test, the Cu atomic flux ($J_{EM}$) was taken to be [44,45,46,47]:

(1) $$J_{EM}=C\frac{D}{kT}F_{e}+C\frac{D}{kT}F_{b}=C\frac{D}{kT}(Z^{*}e\rho j-\frac{\Delta \sigma \Omega }{\Delta x})$$

where C is the concentration of Cu, D is the diffusivity, k is the Boltzmann constant, T is the temperature during the EM test, $F_{e}$ is the electrical force, $F_{b}$ is the mechanical force caused by a stress gradient, $Z^{*}$ is the effective charge number of EM, e is the charge of an electron, ρ is the resistivity of Cu, j is the current density, Δσ is the EM-induced back stress, Ω is the atomic volume, and Δx is the length of metal line.

If $J_{EM}=0$, which means that $F_{e}=F_{b}$, the critical product ${\left(j\Delta x\right)}_{c}$ can be derived, which has been called the short-length effect in Al and Cu stripes [48,49]. The equation of the critical product of current density (j) and length of metal (Δx) is [50]:

(2) $${\left(j\Delta x\right)}_{c}=\frac{\Delta \sigma \Omega }{Z^{*}e\rho }$$

If jΔx is larger than ${\left(j\Delta x\right)}_{c}$, EM damage can occur. In Figure 7, the thickness of the top die RDL is 3 μm and that of the bottom die RDL is 2.4 μm, which makes the current density of the bottom die RDL ($j_{bottom\;RDL})$ larger than that of the top die RDL ($j_{top\;RDL})$. The length of the RDL can be found in Figure 1, Figure 3, and Figure 6. The length of the top die RDL ($\Delta x_{top\;RDL}$) under current stressing is about 125 μm, but that of the bottom die RDL ($\Delta x_{bottom\;RDL}$) is much longer in order to connect the probe pads on the bottom die. Therefore, the product of ${\left(j\Delta x\right)}_{bottom\;RDL}$ is larger than that of ${\left(j\Delta x\right)}_{top\;RDL}$, and the damage of EM is more serious in the bottom die RDL. Of course, higher current density leads to severe Joule heating, but the scale of the RDL and bump and the high thermal conductivity of Cu and Si can reduce the variation in temperature due to Joule heating. 

### 3.5. Much Slower EM Damage in Well-Bonded Samples

**Figure 8:**
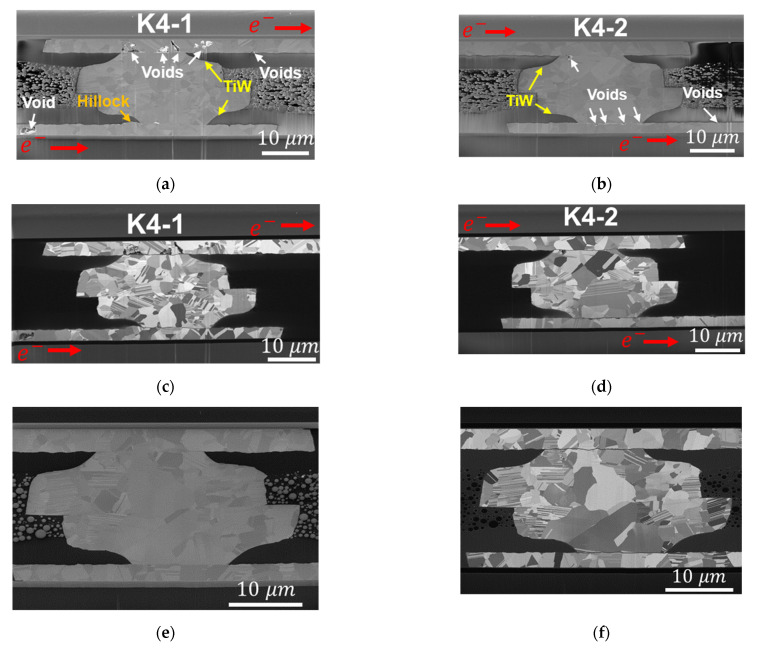
Destructive observation of 90 MPa/30 s_K4 bumps. (**a**,**b**) Electron images of K4-1 and K4-2 bumps, respectively. Voids are observed at the sites of current crowding. The voids are located at the side of electron current passing through the TiW layer. Additionally, voids form in RDLs far from Cu–Cu bumps. (**c**,**d**) Ion images of K4-1 and K4-2 bumps, respectively. The voids caused by EM are located along the grain boundary and TiW layer at the PSV opening. (**e**,**f**) Electron image and ion image of dummy bumps, respectively. No Cu oxide at the surface of Cu–Cu bumps and RDL appeared during EM test.

The Kelvin bumps of 90 MPa/30 s_K4 were the other sample under the long-time (5022 h) EM test. The resistance changes were 17.7% and 21.7% for K4-1 and K4-2, respectively. The cross-sectional images of 90 MPa/30 s_K4 are shown in Figure 8. Voids tended to form at the surface of the RDL, between TiW and the RDL, and between TiW and PSV opening. These failure modes were similar to those for 90 MPa/30 s_K3. However, in Figure 1, the site of K4 is shown to be located away from the edge of the top die, so there was no oxidation on the Cu surface of K4. In addition, a void formed in the bottom die RDL far from the Cu–Cu bump, which is located on the lower left side of Figure 8a.

Another phenomenon caused by EM is the small hillock that formed at the bottom die RDL of K4-1 in Figure 8a. It was the site of electron current flow into the PSV opening, and the TiW layer stopped the Cu atomic flux so that the accumulation of Cu atoms occurred and formed a hillock on the RDL surface. This phenomenon was similar to the EM of metal strips, in which hillocks formed at the anode [51]. Due to the long EM testing time, Cu grains had sufficient time to grow in Cu–Cu bumps. Therefore, in Figure 7 and Figure 8c,d,f, the bonding interfaces changed into zig-zag-type grain boundaries [52], and there were much fewer fine grains than those of the as-fabricated Cu–Cu bumps in Figure 2b.

### 3.6. Finite Element Analysis of EM Tests

**Figure 9:**
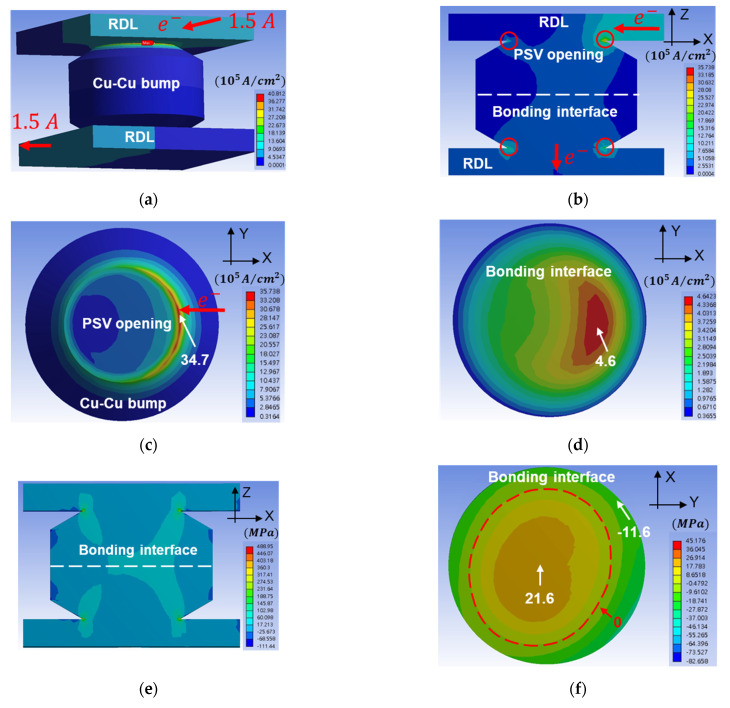
The FEA results of current density distribution and stress distribution. (**a**) The 3D model with electron current direction of single Cu–Cu bump. (**b**) The current density distribution of the cross-section of a Cu-Cu bond. Current crowding is found at the PSV opening, which is marked by red circles. (**c**) The current density distribution at the top die PSV opening. (**d**) The current density distribution at the bonding interface. The maximum current density is $4.6\times 10^{5}\;A/{\mathrm{cm}}^{2}$. (**e**) The distribution of stress along the z-axis at the cross-section of the Cu–Cu bump. (**f**) The distribution of stress along the z-axis at the bonding interface. The maximum tensile stress during the EM test at the bonding interface is 21.6 MPa.

In order to realize the current crowding effect and stress distribution during the EM test, FEA was adopted and is depicted in Figure 9, where the 3D model is shown in Figure 9a. The 1.5 A current was applied, and the cross-section of the Cu–Cu bump is shown in Figure 9b. Current crowding was observed at the edge of PSV opening, which is marked by red circles. The top view of the PSV opening is shown in Figure 9c. The maximum current density was $3.4\times 10^{6}\;A/{\mathrm{cm}}^{2}$, and the shape of the current crowding area was crescent-like. This implies that the void distribution of the PSV opening or RDL was located at the edge of the contact area between the Cu–Cu bump and RDL, and the actual result is shown in Figure 6a, which matched the current crowding area of the FEA result. Figure 9d shows the current density distribution at the bonding interface. The maximum value of current density was $4.6\times 10^{5}\;A/{\mathrm{cm}}^{2}$, so EM will occur slowly.

As the circuit of Kelvin bump A of 31 MPa/10 s_K1 showed an open circuit during the EM test in Figure 5b, the tensile stress distribution at 200 °C was of interest and is shown in Figure 9e. Tensile stress along the z-axis was thought to be the main reason for allowing the Cu–Cu bump to open at the bonding interface. In Figure 9e,f, both sides of the model were set as mirror images to simulate the site of the chip corner, so that the tensile stress distribution is not symmetrical in the Cu–Cu bump. To further emphasize the tensile stress distribution on the bonding interface, Figure 9f shows the tensile stress at the bonding interface. The maximum tensile stress of 21.6 MPa was close to the center of the bonding interface. Interestingly, the edge of the bonding interface was under compressive stress, and the maximum compressive stress was 11.6 MPa. The red dashed line is the zero-stress line along the *z*-axis. Therefore, the bonding strength should be larger than 21.6 MPa in order to sustain the Cu–Cu bump structure during the EM test.

**Figure 10:**
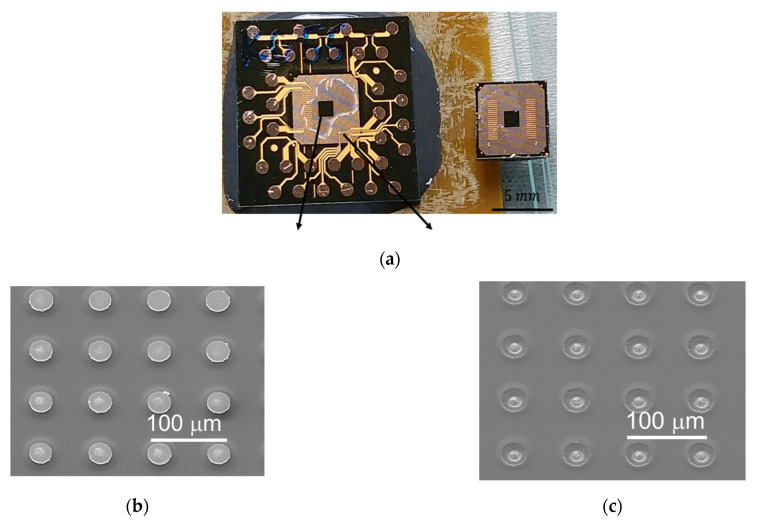
(**a**) The images of the chip after tensile test. The bonding condition is 300 °C/90 MPa/30 s. Two kinds of fracture modes are observed. (**b**) At the center area, fracture tended to form at the bonding interface. (**c**) At the edge of the bump array, fracture occurred at PSV openings.

Bonding strength was measured by the pull test, as given in Table 1, and the fracture mode is shown in Figure 10. The bonding strength was calculated by the pull force divided by the total bonding area. The total bonding area equaled a single bonding interface area multiplied by 4548 bumps, and the value was about 3.2 ${\mathrm{mm}}^{2}$. The bonding strength of bonding conditions 300 °C/90 MPa/30 s was 50.86±3.12 MPa, and that of 300 °C/31 MPa/10 s was 5.25±0.34 MPa. Thus, Kelvin bumps of 31 MPa/10 s_K1 had a high chance of becoming an open circuit. Figure 10 shows the fracture results of bonding conditions at 300 °C/90 MPa/30 s. The center of the array of bumps fractured at the bonding interface, so a ductile fracture surface is shown in Figure 10b. On the other hand, bumps around the corners and edges fractured at the PSV opening, and the ductile fracture at PSV opening is shown in Figure 10c. The white dashed line in Figure 10a shows the distribution of fracture modes in Figure 10b,c. The reason for this kind of distribution might be the process capability, such as chemical mechanical planarization and leveling of the bonding machine.

### 3.7. EM Failure Mechanisms in Cu-Cu Bonding

**Figure 11:**
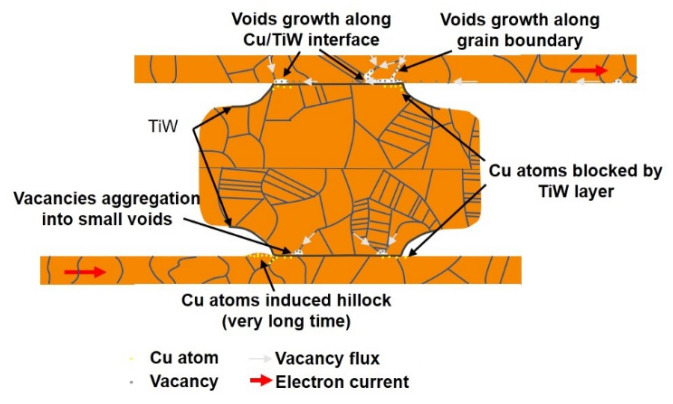
The relationships during the EM test among electron current direction, Cu atoms, vacancies, and TiW layers. The Cu atoms and vacancies are emphasized on the anode and cathode, respectively. The anode and cathode are defined by the electron current direction and the TiW layer. Due to those mechanisms, voids and hillock can be formed.

Based on the experimental data above, the EM failure mechanisms of Cu–Cu direct bonding are discussed below, and the relationships during the EM test among electron current, Cu atoms, vacancies, and TiW layers are illustrated in Figure 11. According to Reference [47], the surface diffusivity on Cu is about two orders of magnitude faster than that of grain boundary diffusivity at 200 °C. Another research report showed that the interface of Cu and a surface coating layer is the other fast diffusion path. Therefore, in this study, surface diffusion, Cu/TiW interface diffusion, and grain boundary diffusion were the three main diffusion paths. The current density of the current crowding effect and RDLs was close to or higher than $10^{6}\;A/{\mathrm{cm}}^{2}$, so EM of Cu will occur at the edge of the PSV opening and RDLs, and only the grain boundaries at those two sites will participate in the EM. Thus, the grain size at the PSV opening and RDLs will influence the EM lifetime, and the interface of Cu/TiW will lead to the void formation along the contact area of the Cu–Cu bump and Cu RDLs. A previous research report showed that Cu atoms cannot diffuse through the TiW layer [41]. Therefore, Cu atom diffusion will be retarded and accumulate at the Cu/TiW interface at the side of electron current flow in the TiW layer. On the other hand, vacancies will accumulate at the side of electron current flow out of the TiW layer, and will aggregate into voids.

The critical product (${\left(j\Delta x\right)}_{c}$) of Equation (2) is related to back stress (Δσ). The back stress (Δσ) can be introduced from the surrounding Cu atoms, confinement of TiW layers, PSV layers, and Si substrates, and the residual stress of electroplating and thermal annealing. However, the stress from the PBO confinement can be ignored for the soft material, and the residual stress can be relaxed during the long EM test time. Thus, the back stress is mainly caused by the surrounding Cu atoms and the confinement of TiW layers. Additionally, the Cu atomic flux of Equation (1) is related to the mechanical force induced by the back stress gradient. If the length (Δx) of Cu circuits is longer, the influence of back stress on Cu EM will be smaller, so the void formation in Cu RDLs is more significant. 

In the Cu RDLs, the PBO layer cannot restrict the Cu hillock formation beside the PSV opening. For an EM test time long enough for Cu atom accumulation, a hillock can be observed in Figure 8a; however, the dimension of RDLs, which is the sink of Cu atoms, is so large that the hillock is not obvious. On the contrary, the accumulation of Cu atoms in Cu–Cu bumps is less for the short length effect, and the TiW layer can restrain any protrusion. Therefore, no hillock can be recognized in the anode of the Cu–Cu bump. Combined with the effects of the diffusion paths, TiW layers, and critical products of the Cu–Cu bump and RDLs, voids are observed at the site of current crowding, and in the RDL, which are depicted in Figure 11.

**Figure 12:**
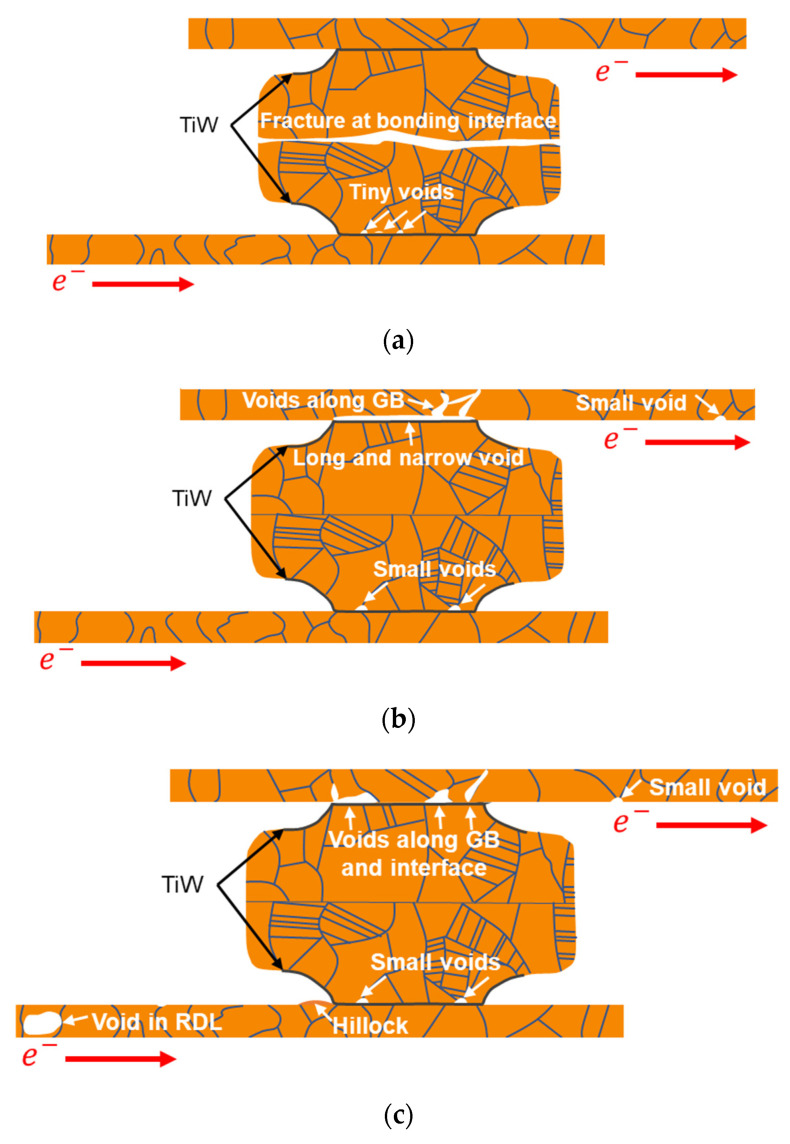
Three kinds of EM failure modes. (**a**) If the bonding strength cannot sustain the tensile stress during the EM test, fracture will occur at the bonding interface. Some tiny voids will form at the PSV opening due to the current crowding effect. This is a kind of early failure. The Cu–Cu bumps of bonding condition 300 °C/31 MPa/10 s with the bonding strength of about 5 MPa will lead to this failure mode. (**b**) A long and narrow void at the contact area of the PSV opening and RDL, which leads to an open circuit. Some voids grow along grain boundaries. Some small voids form in the Cu–Cu bump at the bottom PSV opening and RDL surface. The Cu–Cu bumps of the bonding condition 300 °C/90 MPa/30 s with the bonding strength of about 50 MPa have a chance to cause this failure mode. (**c**) Under an excellent bonding condition, some void formation may occur along grain boundaries and the interface in the RDL. Small voids form at the bottom PSV opening of the Cu–Cu bump and RDL surface. In addition, void formation occurs randomly in the RDL far from the Cu–Cu bump. The Cu–Cu bumps of bonding condition 300 °C/90 MPa/30 s with a bonding strength of approximately 50 MPa have an opportunity to bring about this failure mode.

Considering the Cu atomic diffusion paths and the bonding strength, three kinds of EM failure mode are shown in Figure 12. In Figure 12a, if the bonding strength is not strong enough, Cu–Cu bumps have a high chance to fracture at the bonding interface. This is the early failure mode. Due to the short EM test time, voids at the PSV opening tend to be tiny and form at triple junctions of the grain boundary and Cu/TiW interface. 

In Figure 12b, a long and narrow void formed at the passivation contact area between the Cu–Cu bump and RDL. When the current density or testing temperature is severe, this failure mode will become another killer of EM lifetime. Future work might involve optimizing the process to find an excellent adhesion layer to strengthen the interface of the Cu/adhesion layer [53]. Due to the short-length effect in EM, the void size in the RDL is larger than that in the Cu–Cu bump. Some small voids might randomly form at the surface of the RDL for the long EM lifetime. 

In Figure 12c, if voids at the contact area between the Cu–Cu bump and RDL do not merge together, the EM lifetime will be much longer. Voids also tend to form at the grain boundary and surface, and the void size is influenced by the short-length effect. Besides, both void and hillock formation can occur under such a long EM lifetime, over 5000 h.

## 4. Summary

Three kinds of EM failure mechanisms are summarized below. First, if the bonding strength is not strong enough, the circuit of the Cu–Cu bump will open and lead to early failure. Second, if the bonding strength can sustain the tensile stress from the thermal mismatch of materials, EM–induced Cu diffusion and vacancy diffusion can last for a long time, until the growth of voids along the passivation interface leads to failure. Third, provided that no fracture occurs at the bonding interface, as well as at the passivation interface because of an excellent bonding process, a very long lifetime of electromigration, over 5000 h, can be obtained, even though some small voids may have formed on the surface or interface in the bonding microstructure. However, if the diameter of the Cu–Cu bump continuously shrinks below 10 μm, the current density must become larger and the EM of the Cu–Cu bump will become a critical issue.

## Figures and Tables

**Table 1:** Bonding strength of two bonding conditions.

Bonding Condition	Bonding Pressure (MPa)
300 °C/90 MPa/30 s	50.86±3.12
300 °C/31 MPa/10 s	5.25±0.34

## Data Availability

Not applicable.
